# Cardiac Arrest as the First Manifestation of Single Coronary Artery—Potential Role of FFR_CT_ for Detecting Critical Flow Reduction. A Case Report

**DOI:** 10.1002/ccr3.72918

**Published:** 2026-06-22

**Authors:** Frantisek Nehaj, Robert Hatala, David Kocan, Carlos Collet, Peter Hlivak, Martin Svetlosak, Usman Ali, Derek Leslie Connolly

**Affiliations:** ^1^ Department of Arrhythmia and Cardiac Pacing National Cardiovascular Institute Bratislava Slovakia; ^2^ Faculty of Medicine and National Institute of Cardiovascular Diseases Bratislava, Clinic of Arrhythmology Comenius University Bratislava Faculty of Medicine Bratislava Slovakia; ^3^ Department of Diagnostic and Interventional Radiology National Cardiovascular Institute Bratislava Slovakia; ^4^ Department of Radiology Comenius University Bratislava Faculty of Medicine Bratislava Slovakia; ^5^ Cardiovascular Center Onze‐Lieve‐Vrouwziekenhuis, OLV Clinic Aalst Belgium; ^6^ Department of Cardiology Royal Albert Edward Infirmary Wigan UK; ^7^ Midland Metropolitan University Hospital (MMUH) Birmingham UK; ^8^ Institute of Cardiovascular Sciences University of Birmingham Birmingham UK

**Keywords:** cardiac arrest, coronary artery anomaly, fractional flow reserve—Computed tomography, single coronary artery, ventricular fibrillation

## Abstract

Single coronary artery may present with life‐threatening ventricular arrhythmias even in the absence of structural heart disease or inducible ischemia. FFR‐CT can detect clinically relevant flow reduction despite normal functional testing, highlighting its potential role in risk stratification in patients with coronary anomalies.

**Abbreviations**
ACS: acute coronary syndrome
CAA: coronary artery anomaly
CAAs: coronary artery anomalies
CAD: coronary artery disease
CCTA: cardiac computed tomography angiography
CMR: cardiac magnetic resonance
CX: circumflex
FFR‐CT: fractional flow reserve computed tomography
ICD: implantable cardioverter defibrillator
RCA: right coronary artery
SCD: sudden cardiac death
SPECT: single photon emission computed tomography
VF: ventricular fibrillation

Undiagnosed coronary artery anomaly (CAA) is the second most common cause of SCD in young competitive athletes, and myocardial ischemia is considered the primary cause of underlying malignant arrhythmias. Cardiac computed tomography (CCT) has become increasingly important not only for detecting coronary artery disease (CAD) but also for the diagnosis of coronary anomalies. However, the prognostic impact of CAA is highly individual and difficult to predict. In this case, FFR_CT_ was performed as a novel means of evaluating the hemodynamic significance of abnormal coronary circulation due to CAA in a male victim of aborted sudden arrhythmic death.

A 31‐year‐old male was admitted to the hospital after an aborted cardiac arrest. Ventricular fibrillation (VF) occurred during sleep under resting conditions which was diagnosed on‐site and terminated with a single DC shock. Emergency coronary angiography revealed complete right coronary atresia. Thereafter, the patient was referred to our center without neurological sequelae for further management. Echocardiogram and cardiac MRI were normal. An implantable cardioverter‐defibrillator (ICD) for secondary prevention was implanted. Subsequently, the patient underwent complementary investigations to detect myocardial ischemia during rest and exercise—treadmill stress test and single‐photon emission computerized tomography (SPECT) were all within normal limits. In order to assess the hemodynamic impact of the right coronary atresia, CCT with FFR_CT_ calculation (HeartFlow Inc., Redwood City, California, USA) was applied and demonstrated significant flow reduction in the mid to distal segments of the LAD and CX arteries.

This case report demonstrates the potential of CCT and FFR_CT_ to detect a potentially malignant reduction of coronary flow in distal segments of an otherwise normal left coronary tree in the presence of right coronary artery atresia.

## 1 Introduction

Coronary artery anomalies (CAAs) are a group of congenital conditions characterized by abnormal origin, course, or termination of any of the three main epicardial coronary arteries [1]. The increasing use of invasive and non‐invasive cardiovascular imaging detected a 7.9% prevalence of CAAs [2]. CAAs are extremely variable necessitating a highly individualized therapeutic approach [3, 4].

However, clinical correlates and prognostic implications of CAAs remain poorly understood [1, 3, 4]. According to current knowledge, isolated congenital coronary atresia should be considered potentially fatal, increasing the risk of neonatal death and later in the life of heart failure or malignant arrhythmias [5, 6]. Angina, dyspnoea, and chest discomfort may represent the first clinical manifestations. Most importantly, however, CAA‐related cardiac arrest may occur in up to 50% of previously asymptomatic patients [7, 8].

## 2 Case Presentation

A 31‐year‐old male was admitted to the hospital after out‐of‐hospital cardiac arrest followed by 10 min of cardiopulmonary resuscitation performed by the patient's wife who is a non‐healthcare professional. ECG at the arrival of the ambulance confirmed VF, and one successful DC shock was delivered on‐site. The patient was free of cardiovascular risk factors. An urgent coronary angiogram ruled out acute coronary syndrome (ACS), and coronary artery disease was detected; however, right coronary atresia was confirmed. Further cardiac investigations including echocardiography, CMRI, and CCTA were performed to confirm the diagnosis and to rule out other possible causes of VF.

## 3 Investigations

### 3.1 Twelve‐Lead Electrocardiogram (ECG)

Sinus rhythm with no ischemic changes.

### 3.2 Blood Tests

High‐sensitivity Troponin I level was 13 ng/L (normal range < 15 ng/L). Subsequent repeat measurement of Troponin I at 3 h was 10 ng/L; full blood count and renal function tests were normal.

### 3.3 Cardiac Computed Tomography Angiography (CTCA)

Atresia of the right coronary artery (RCA).

### 3.4 CT Fractional Flow Reserve (FFRCT)

Significant flow reduction of CX from mid to distal region with a FFRCT value of less than 0.70.

### 3.5 Echocardiogram

Normal left ventricle systolic and diastolic function, no significant valve and regional wall abnormalities with normal right heart anatomy and function.

### 3.6 Cardiac Magnetic Resonance

Normal biventricular function, no LGE or other evidence of arrhythmogenic substrate.

### 3.7 Single‐Photon Emission Computerized Tomography (Stress Test)

Summed stress scores of less than 3, normal.

### 3.8 Treadmill Exercise Stress Test

No inducible ischemia or angina symptoms.

## 4 Treatment

**FIGURE 1:**
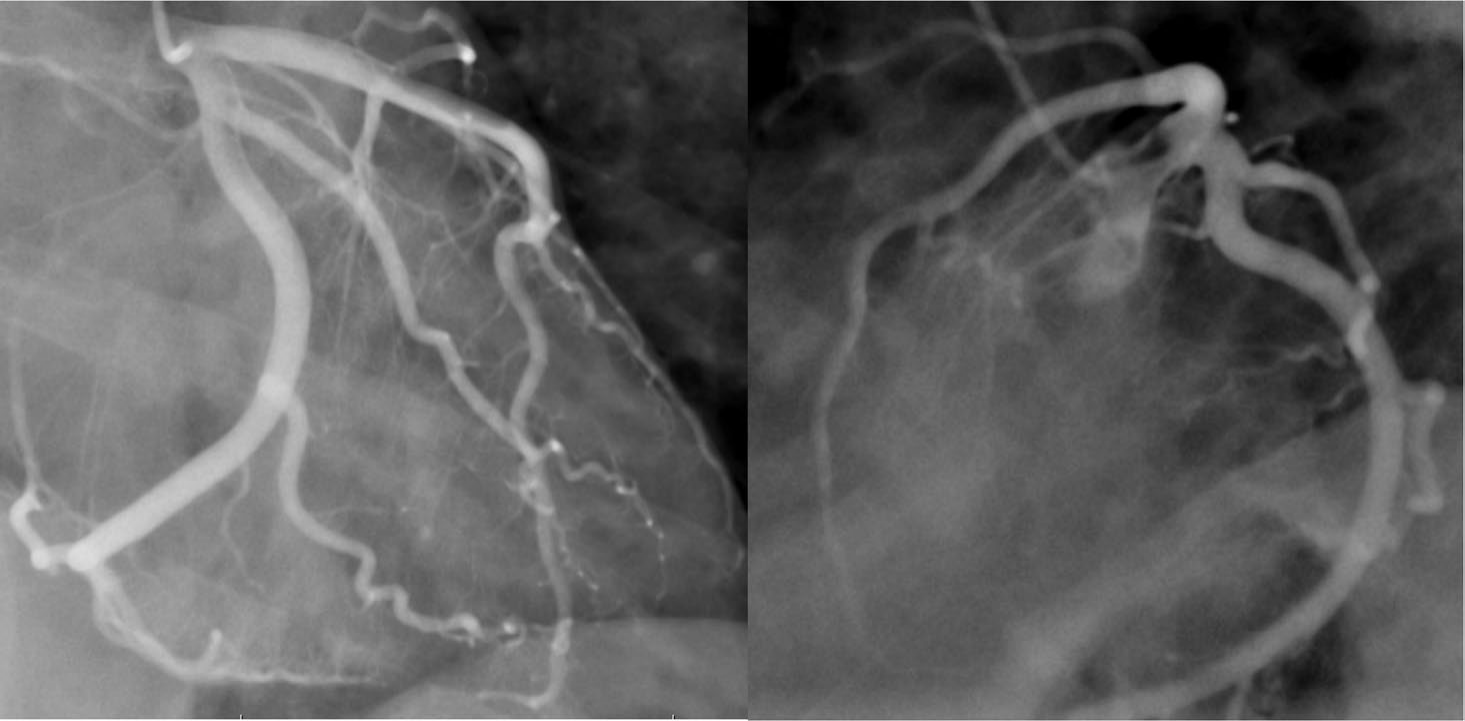
Invasive angiogram—CAA.

**FIGURE 2:**
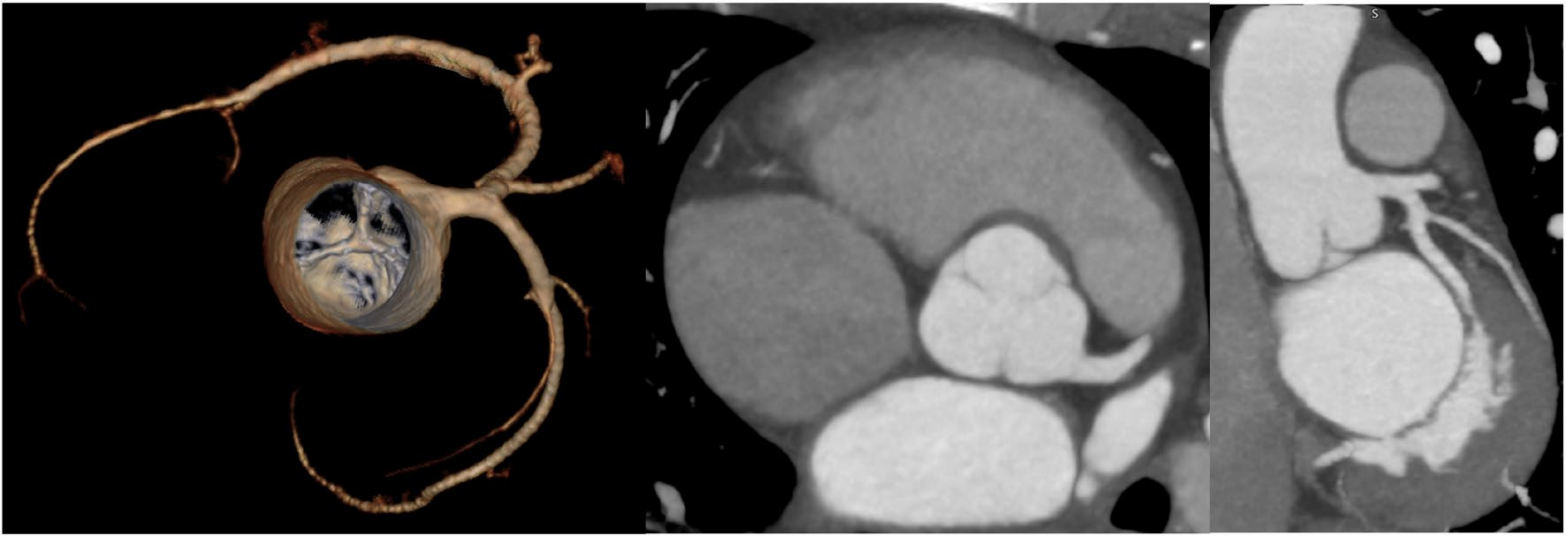
CTCA—RCA atresia.

**FIGURE 3:**
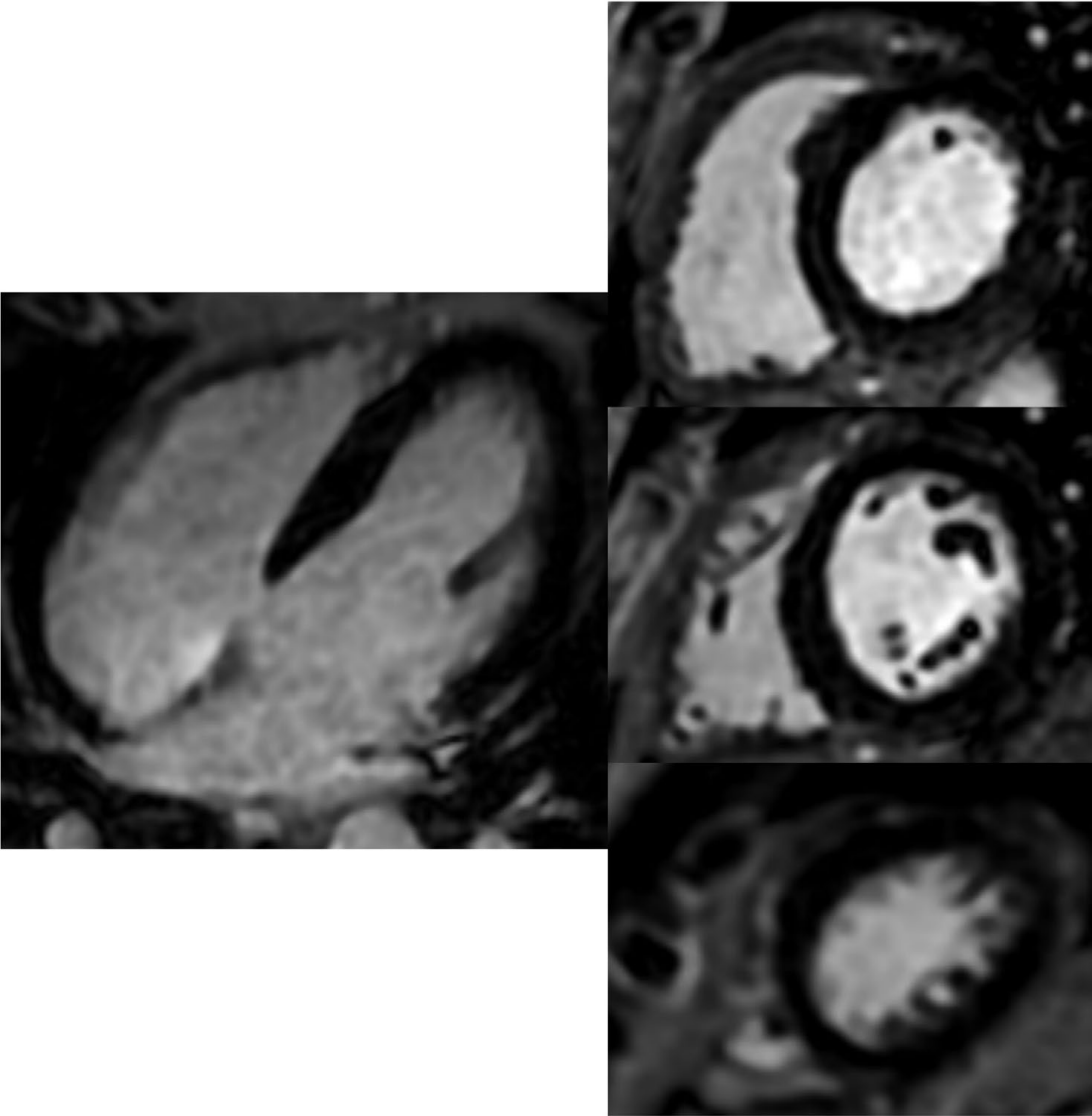
CMRI.

**FIGURE 4:**
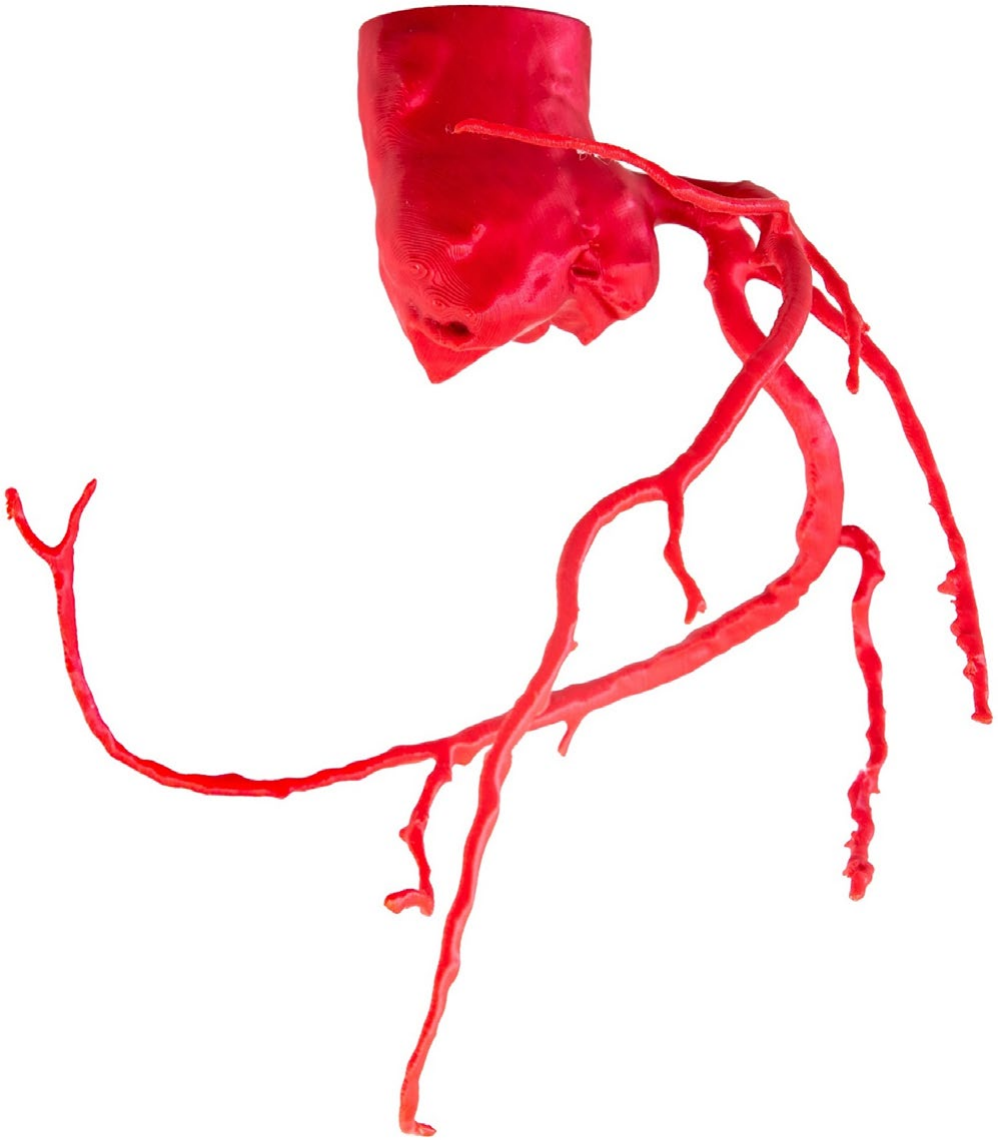
3D printed model of coronary tree.

**FIGURE 5:**
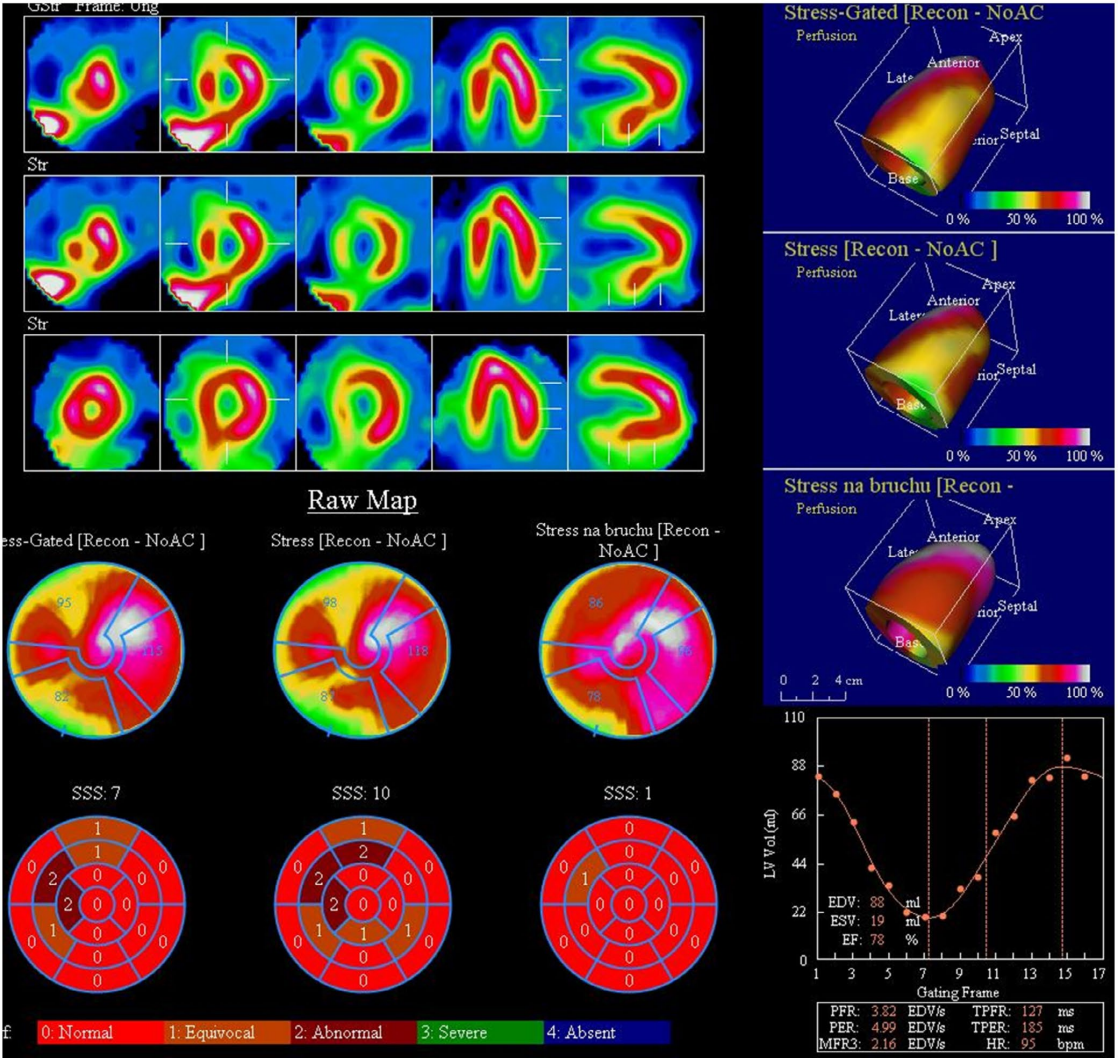
SPECT.

An immediate invasive coronary angiogram should be considered after cardiac arrest due to confirmed VF (Figure 1). The patient underwent urgent invasive coronary angiography with no evidence of coronary artery disease or ACS. However, angiogram and CTCA confirmed RCA atresia (Figures 2 and 3). CMRI (Figure 4) and echocardiography showed no structural heart disease. In light of these findings, ICD for secondary prevention was implanted and the patient was subsequently discharged without drug treatment. Six weeks after ICD implantation, functional testing with SPECT (Figure 5) and treadmill exercise stress test was performed, all with normal findings [9].

**FIGURE 6:**
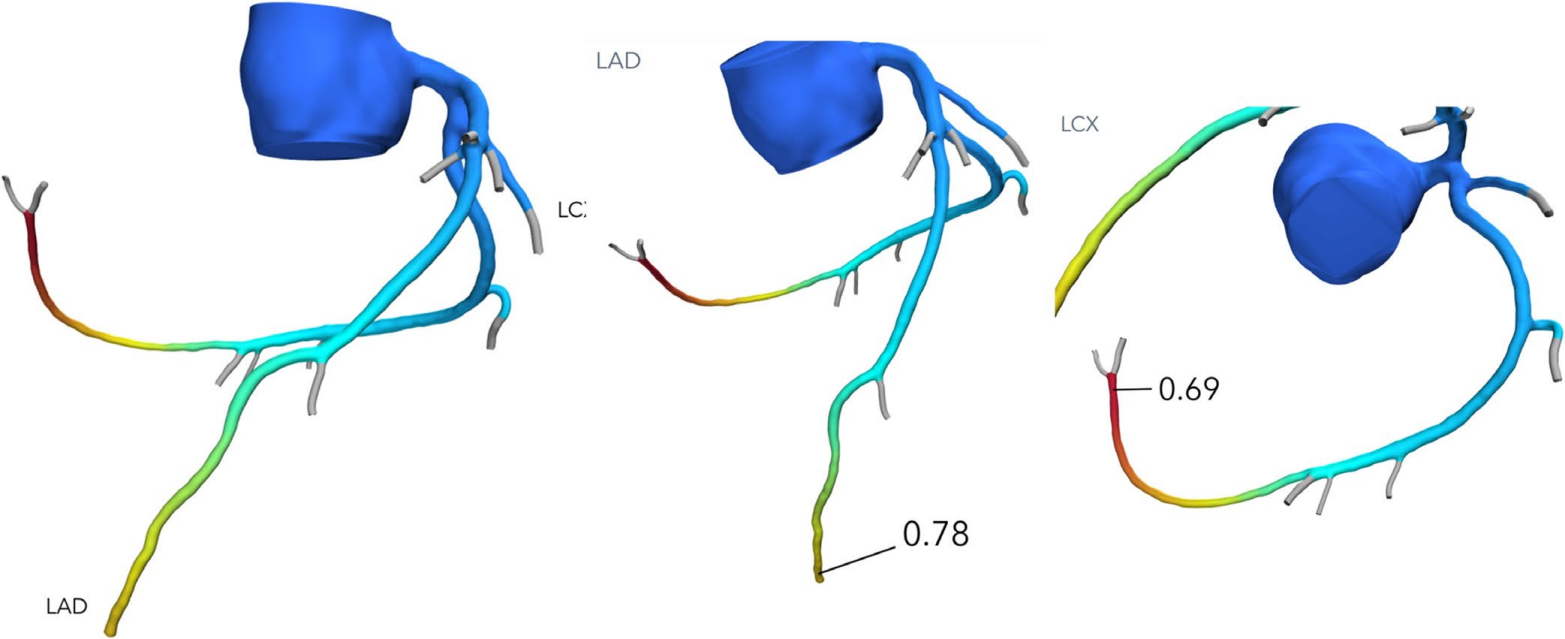
FFR‐CT.

**FIGURE 7:**
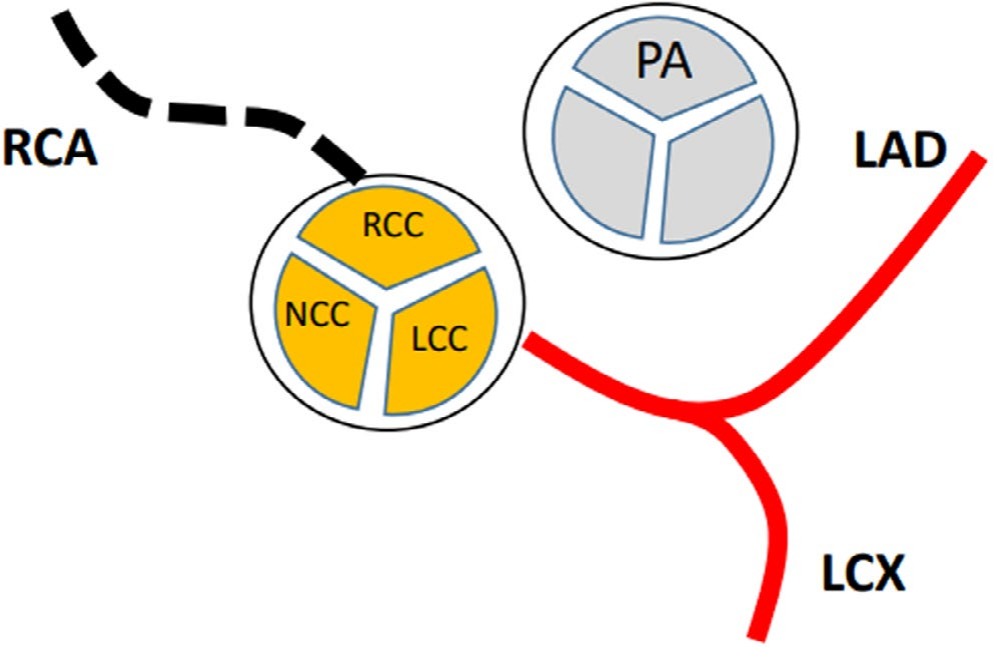
Schematic illustration of the coronary artery anatomy and anomaly.

FFR_CT_ was calculated and a value < 0.70 was detected in mid‐ to distal LAD and CX segments, thus confirming hemodynamically significant flow reduction (Figure 6). The patient was followed up in the cardiology outpatient clinic after 3 months with no further recurrences of malignant arrhythmias. A schematic representation of the coronary anatomy is depicted in Figure 7, demonstrating a normal left coronary artery origin from the left coronary cusp with bifurcation into the left anterior descending and left circumflex arteries, while the right coronary artery lacks a normal ostial origin from the right coronary cusp, consistent with right coronary artery atresia.

## 5 Discussion

FFR_CT_ is used to noninvasively assess the hemodynamic relevance of coronary artery stenoses. It uses CTA images and is defined as the ratio of maximal flow through a stenotic coronary artery to the hypothetical flow achievable in the same coronary artery if it was normal [10]. This method enables the identification of flow‐limiting coronary lesions that may cause myocardial ischemia. The use of FFR_CT_ in CAAs has not been systematically evaluated [11, 12]. Performing additional myocardial perfusion imaging tests (SPECT, treadmill exercise stress test) did not detect ischemia. In this case, FFR_CT_ appears to better predict the hemodynamic significance of right coronary atresia.

CAAs with anomalous aortic origin have been associated with SCD in young athletes as the second most common cause [13, 14]. Most of the available studies have focused on young individuals practicing strenuous exertion. The prognostic impact of CAAs in nonathletes or in the elderly remains obscure. The single coronary artery presenting with VF is limited to very few real‐life case reports [15]. Our case report is unique due to the fact that VF occurred in a non‐athlete during sleep in a bed. Luckily, arrhythmic death was aborted by early layperson resuscitation and early defibrillation.

Little is known about the use of serial invasive or non‐invasive FFR measurements to evaluate CAAs in clinical practice and this case report suggests its possible role in detecting insufficient coronary flow. Current guidelines recommend conservative management except for ICD implantation for secondary prevention [1, 4].

The etiology of VF in this case is likely multifactorial. Although chronic myocardial ischemia related to altered coronary flow in the setting of a single coronary artery represents a plausible mechanism, alternative contributors should be considered. In particular, autonomic imbalance, coronary vasospasm, idiopathic and sleep‐related triggers may have played a role in arrhythmia initiation [16, 17, 18, 19, 20]. These factors could not be definitively excluded. However, comprehensive evaluation including electrocardiography, cardiac magnetic resonance imaging, and the absence of structural heart disease reduces the likelihood of a primary electrical or structural substrate. Therefore, chronic ischemia due to abnormal coronary anatomy should be regarded as a probable, rather than definitive, cause of VF in this patient.

FFR_CT_ has not yet been validated against invasive FFR in patients with coronary anomalies, which represents a significant gap in our research [11, 12, 21, 22, 23]. It's important to emphasize that FFR_CT_ is a lesion‐specific ischemia test, distinct from tests that assess myocardial or artery‐specific ischemia. A major concern is that FFR_CT_ often produces ‘pathological’ values when measured at the most distal segments of the arteries, although the right coronary artery (RCA) may be an exception. This inconsistency underscores the necessity of measuring FFR_CT_ 10 to 20 mm distal to the end of a lesion to ensure more accurate readings. In this particular case, FFR_CT_ was measured correctly in both the distal and mid sections of the arteries, revealing ischemia.

FFR‐CT is a non‐invasive method for assessing the hemodynamic relevance of coronary lesions; however, it has important limitations that must be considered. Its accuracy depends on several physiological assumptions and may be influenced by factors such as intraventricular pressure and vessel geometry, particularly in distal coronary segments where measurements can be more variable. In contrast, invasive fractional flow reserve (FFR) using a pressure wire remains the gold standard for functional assessment of coronary flow. In the present case, the interpretation of FFR‐CT is further complicated by discordant findings from functional testing, as both SPECT and treadmill exercise testing were normal. Therefore, the FFR‐CT results should be interpreted with caution, and their clinical significance remains uncertain in this specific context [24, 25].

However, it is noteworthy that this case report presents conflicting results, showing a normal SPECT outcome alongside an abnormal FFR_CT_.

## 6 Conclusion

Single coronary arteries represent potentially life‐threatening anomalies, especially in the young population, and are most often discovered only post‐mortem or as an incidental finding during investigations for other cardiac or noncardiac reasons. This report suggests that FFR_CT_ might be useful for detecting inadequate flow in the otherwise normal remaining arteries even in cases where SPECT and treadmill exercise stress tests did not detect myocardial ischemia. This information might be of value for individualized risk stratification.

## Author Contributions

**Frantisek Nehaj:** conceptualization, investigation, writing – original draft, writing – review and editing. **Robert Hatala:** supervision, writing – review and editing. **David Kocan:** investigation, writing – review and editing. **Carlos Collet:** investigation, supervision, writing – review and editing. **Peter Hlivak:** writing – review and editing. **Martin Svetlosak:** writing – review and editing. **Usman Ali:** writing – review and editing. **Derek Leslie Connolly:** supervision, writing – review and editing.

## Funding

This work was supported by Univerzita Komenského v Bratislave.

## Consent

The authors confirm that written consent for submission and publication of this case report including images and associated text has been obtained from the patient in line with COPE guidance.

## Conflicts of Interest

The authors declare no conflicts of interest.

## Data Availability

All data relevant to the case are included in this published article. No additional datasets were generated or analyzed during the current study. Further information is available from the corresponding author upon reasonable request, subject to patient confidentiality requirements.
